# Thyroid Hormone Distributor Proteins During Development in Vertebrates

**DOI:** 10.3389/fendo.2019.00506

**Published:** 2019-08-08

**Authors:** Sarah A. Rabah, Indra L. Gowan, Maurice Pagnin, Narin Osman, Samantha J. Richardson

**Affiliations:** School of Health and Biomedical Sciences, RMIT University, Melbourne, VIC, Australia

**Keywords:** albumin, development, evolution, phylogeny, thyroid hormones, thyroxine-binding globulin, transthyretin, vertebrates

## Abstract

Thyroid hormones (THs) are ancient hormones that not only influence the growth, development and metabolism of vertebrates but also affect the metabolism of (at least some) bacteria. Synthesized in the thyroid gland (or follicular cells in fish not having a discrete thyroid gland), THs can act on target cells by genomic or non-genomic mechanisms. Either way, THs need to get from their site of synthesis to their target cells throughout the body. Despite being amphipathic in structure, THs are lipophilic and hence do not freely diffuse in the aqueous environments of blood or cerebrospinal fluid (in contrast to hydrophilic hormones). TH Distributor Proteins (THDPs) have evolved to enable the efficient distribution of THs in the blood and cerebrospinal fluid. In humans, the THDPs are albumin, transthyretin (TTR), and thyroxine-binding globulin (TBG). These three proteins have distinct patterns of regulation in both ontogeny and phylogeny. During development, an additional THDP with higher affinity than those in the adult, is present during the stage of peak TH concentrations in blood. Although TTR is the only THDP synthesized in the central nervous system (CNS), all THDPs from blood are present in the CSF (for each species). However, the ratio of albumin to TTR differs in the CSF compared to the blood. Humans lacking albumin or TBG have been reported and can be asymptomatic, however a human lacking TTR has not been documented. Conversely, there are many diseases either caused by TTR or that have altered levels of TTR in the blood or CSF associated with them. The first world-wide RNAi therapy has just been approved for TTR amyloidosis.

## Thyroid Hormones

Thyroid hormones (THs) are fundamentally involved in the regulation of growth, development, and overall metabolism, particularly of the CNS. Despite being amphipathic in structure, THs are lipophilic compounds and readily partition between the lipid phase and the aqueous phase with a ratio of about 20,000:1 (1). Therefore, THs are not freely diffusible in the aqueous environments of the blood and cerebrospinal fluid (CSF).

THs are considered evolutionarily “old” hormones, as THs and their derivatives impact the metabolism of not only vertebrates but also bacteria (2), ascidians, tunicates, and other invertebrate species [for review see Holzer et al. (3)]. Furthermore, the endostyle of tunicates can incorporate iodine into tyrosine residues which are then incorporated into proteins, rendering the endostyle the functional precursor (from a TH perspective) to the thyroid gland (4). In amphibians, reptiles, birds, and mammals, THs are synthesized in the thyroid gland, which is a discrete gland located at the base of the neck. In fish, however, the shape and location of the thyroid gland varies considerably between species e.g. diffuse follicles around the ventral aorta (cyclostomes), or near the branchial arteries of the gills (some teleosts) or a compact gland near the branchial arch (elasmobranchs) (5).

**Figure 1:**
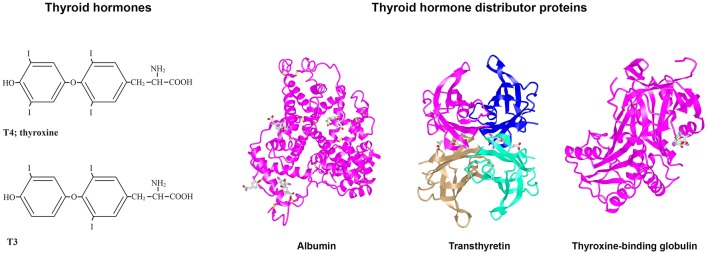
Structures of T4, T3, albumin, TTR, and TBG. **(Left)** Structures of T3 and T4. **(Right)** 3D protein structures and T4 binding sites of the three THDPs in humans: albumin (7), transthyretin (8), and thyroxine-binding globulin (7–10).

Following synthesis in the thyroid gland (or follicles, as for fish), the THs are secreted via TH transmembrane transporters into the blood. In vertebrate species studied to date, most TH secreted by the thyroid is in the form of 3,3′,5,5′-tetraiodo-L-thyronine (thyroxine; T4) and less is in the form of 3,3′5-triiodo-L-thyronine (T3) (6) (see Figure 1). Due to the lipophilicity of THs (as mentioned above), they preferentially partition into the lipid environment of membranes (11). However, this can be counteracted with the presence of plasma proteins that bind THs and enable distribution of the THs from their site of synthesis to their target cells throughout the body. Thus, these proteins are termed “TH Distributor Proteins” (THDPs) (12). In humans, the THDPs are albumin, transthyretin (TTR), and thyroxine-binding globulin (TBG) (see Figure 1). Greater than 99.7% of THs in the blood of mammals is bound to THDPs, rendering a very small fraction in the free form. Because only the free (non-protein bound) THs can enter cells, it is very important that the THDPs regulate the amount of free THs in the blood (and CSF).

## Thyroid Hormone Distributor Proteins (THDPs)

In humans, the main THDPs are albumin, TTR, and TBG. Albumin is a single polypeptide chain protein with a molecular mass of about 67 kDa that is rich in alpha helical structure (see Figure 1). At physiological pH, albumin has a heart shape globular structure but under acidic pH adopts an elongated “cigar conformation” (these conformational changes are reversible). Albumin comprises about half the total protein in blood (~40 g/l) and can bind many compounds weakly: THs, fatty acid, drugs etc. (13). TTR is a 55 kDa homotetrameric protein rich in beta sheet structure and can be considered a dimer of dimers which come together with a central channel that has two TH binding sites [Blake et al. (14); see Figure 1], although under physiological conditions only one site is filled due to negative co-operativity (9). TTR is present in healthy adult blood at about 0.25 mg/l and can also bind up to two molecules of retinol-binding protein, which in turn bind retinol (15). Thus, TTR distributes two ligands for nuclear hormone receptors. TBG is a 54 kDa monomeric protein that has only a single site for TH binding and is highly glycosylated (see Figure 1). Whilst due to its structure TBG can be considered a serpin (serine protease inhibitor), it does not actually function as a serpin. Albumin, TTR, and TBG each have differing affinities and on/off rates for T4 and for T3 [for greater details and discussion, see (16)]. In general, albumin binds quite weakly, TTR has intermediate affinity and TBG has the highest affinity for THs. In mammals, each of these three THDPs binds T4 with higher affinity than T3. This provides a buffering like system, for maintaining the free level of THs [see (12)]. The range is from the concentration of free T4 in blood up to the maximum solubility of T4 at pH 7.4. Some text books claim that the reason for hydrophobic signal molecules being bound to protein in the blood is due to poor solubility of the hydrophobic signal compound in the blood, but this is not true. The maximum solubility of T4 at pH 7.4 is 2.3 μM (17) i.e., 100,000 times the concentration of free T4 in human blood (24 pM). The function of THDPs in enabling TH distribution was shown by a set of elegant experiments by Mendel et al. (11): rat livers perfused with T4 in the absence of THDPs resulted in T4 partitioning into the first cells they came in contact with; whereas when livers were perfused with T4 together with THDPs, this resulted in a uniform distribution of T4 throughout the liver and T4 also in the perfusate.

Albumin and TBG have higher affinity for T4 than for T3 in all species studied. However, this is not the case for TTR: in all studied species of birds, reptiles, amphibians, and fish [with the notable exception of sea bream, where TTR had similar affinity for T3 and T4 (18)],TTR has higher affinity for T3 than for T4 (19–24). Only in mammals, does TTR have higher affinity for T4 than for T3 [for a detailed discussion on how and why this may have occurred during evolution, see (25)].

## Distribution of THDPs in Adult Vertebrates

Not all vertebrates have all three THDPs in their blood. There are clear patterns based on various groups of vertebrates, during both ontogeny and phylogeny. The traditional way of identifying THDPs in blood of various species was analyzing serum or plasma directly for radioactively-labeled T3 or T4 binding to proteins. The discussion in this paragraph relates to data collected in that way. Of the ~150 species of adult vertebrates studied, all had albumin as a THDP in their blood (26–30). For some groups of animals, albumin was the only THDP. In general: fish, amphibians, and reptiles and monotremes (echidna and platypus) and some polyprotodont marsupials. For another set of animals, both albumin and TTR were present in blood. In general: birds, diprotodont marsupials, and some eutherians (“placental mammals”). The final set had all three THDPs. This group only comprised some eutherian mammals but we could not discern a clear pattern within eutherians for presence/absence of TBG [previous studies had suggested TBG was present in “larger mammals” but this no longer holds true e.g., (28)]. In general, there has been an increase in TH distribution capacity during vertebrate evolution, both in the number of THDPs and in light of each “new” THDP having higher affinity for THs than the previous i.e., albumin (original THDP with weak affinity for THs) then TTR (second THDP appearing during evolution, with higher affinity for THs than albumin) then TBG [third THDP appearing, with higher affinity for THs than TTR; see (31)].

More recently, with the ever-expanding number of genomes and transcriptomes that are accessible via publicly available databases, it has been possible to identify genes and mRNA or expressed sequence tags (ESTs) corresponding to proteins of interest. This approach can be valuable for identification of low abundance proteins. Whilst this can be an alternative approach to identification of proteins being synthesized in a given tissue of a particular species at a defined stage of life, this can also generate many “false positives” if only considered superficially. It is very time-consuming to check each transcript for complete integrity (full length, lack of internal stop codons etc.). Never the less, this approach has been used successfully to identify TTR in many species of fish (32). Given the huge diversity of fish (>25,000 species), the data presented in Table 1 that were collected the “traditional” way, are probably overly simplistic and not truly representative of the actual situation in nature. “Omics” approaches are required to further investigate the distribution of TTR synthesis in each group of fish and the corresponding ontogenic analyses. Furthermore, the variation in piscine TTRs to date has already revealed that some fish TTRs bind T3 with higher affinity than T4 whereas others bind both ligands with similar affinity (see above).

**Table 1:** Vertebrate species with additional THDP with higher affinity for TH in blood during development.

	**Development**	**Adult**	**Reference (for each species)**
**Fish:** *Sparus aurata Oncorhynchus massou Salmo salar Oncorhynchus tshawytscha*	TTR Albumin	Albumin	(22, 33) (34) (35) (35)
**Amphibians:** *Xenopus laevis* *Rana catesbeiana*	TTR Albumin	Albumin	(36) (20)
**Reptile:** *Crocodylus porosus*	TTR Albumin	Albumin	(35)
**Mammals**			
Marsupials—Polyprotodonta: *Sminthopsis crassicaudata*	TTR Albumin	Albumin	(35)
Diprotodonta: *Macropus eugenii*	TBG TTR Albumin	TTR Albumin	(35) (37)
Eutherians—Rodentia: *Rattus norvegicus* *Mus musculus*	TBG TTR Albumin	TTR Albumin	(38) (39)

*Adapted from Richardson et al. (35)*.

For those animals with three THDPs, TTR is responsible for most of the delivery (bioavailability) of T4 (40). This is in contrast to the previously held belief that because TBG binds about 75% of T4 in blood (albumin binds about 10% and TTR binds about 15%), that TBG is responsible for the delivery of T4. We think of the situation as analogous to Goldilocks and the Three Bears (41): albumin binds so weakly that it is not very efficient in distributing T4; TBG binds so tightly that it is not efficient in releasing T4 (more like a storage reservoir in the blood); but due to the combination of off rates and capillary transit times, TTR is responsible for most of the delivery of T4 to tissues (42).

## THDPs in Blood During Vertebrate Development

The pattern of THDPs in blood during development differs from that in adults (see below). In general, it was revealed that there was an additional THDP present during specific stages of development, compared to adulthood. For example, in two species of salmon, where albumin is the only THDP in adults, TTR was also present at smoulting (35) and in juvenile fish (22, 33, 34). Whereas, adult amphibian had only albumin as a THDP in blood, around the time of metamorphosis TTR was also present (20, 23, 36). Juvenile saltwater crocodiles were found to have TTR in addition to albumin, and the polyprotodont marsupial (fat-tailed dunnart) had TTR in addition to albumin during development (35). The diprotodont marsupial tammar wallaby had a TBG-like protein during development in addition to the albumin and TTR present in adults (37). All vertebrates have a transient surge in TH levels at a specific stage in development (6). The additional THDPs appear to coincide with the elevated TH levels in blood (35). This provides an augmented TH distribution capacity at the time when TH levels in blood are elevated during development (see Table 1). In this table, we consider only plasma proteins. However, whilst plasma proteins are responsible for the majority of TH distribution in homeotherms, some fish are known to have the bulk of their THs distributed in the blood by lipoproteins (43) and a small proportion of THs in human blood (44). Oviparous animals (including some fish, amphibians, reptiles, birds, monotremes, and some invertebrates) also synthesize another lipoprotein: vitellogenin, an egg yolk precursor protein synthesized in the liver. Vitellogenin is a TH binding protein. In females, vitellogenin levels cycle according to estrogen levels. However, TH can regulate the levels of vitellogenin by inducing estrogen receptor alpha (45).

## THDPs in the CSF

THs must cross the blood-brain barrier or the blood-CSF barrier in order to enter the CNS. These two barriers have been studied most extensively in mammals but some information is known about other vertebrates also. However, to the best of our knowledge, the quantitative contribution of each pathway (crossing the blood-brain barrier or crossing the blood-CSF barrier, relative to the other) for TH entering the brain is not yet known.

The blood-CSF barrier is formed by the tight junctions between the epithelial cells of the choroid plexus. The choroid plexus is a villous structure located in the lateral, third and fourth ventricles of the brain and is responsible for secreting about 70% of the CSF (46). In adults, the major protein synthesized and secreted by the choroid plexus from studied species of mammals (eutherians, marsupials, and monotremes), birds, and reptiles is TTR (24, 29, 47–50). This TTR is secreted toward the CSF and not into the blood (51) and has been implicated in moving T4 (but not T3) from the blood across the choroid plexus into the CSF (1, 52, 53). The major protein synthesized and secreted by the choroid plexus of amphibians is a lipocalin, specifically: prostaglandin D synthetase (54, 55) also known as Cpl1 (55) and β-trace (56). This protein has a calyx structure which could be used for binding small hydrophobic molecules and thus could have been a functional precursor to TTR, although possibly not transporting THs.

## TTR Gene Expression in the Choroid Plexus During Development

In those species studied, the choroid plexus has the highest concentration of TTR mRNA per tissue weight compared to other tissues in the body e.g., 11- to 22-fold higher than in the liver [see (57)]. However, the timing for the maximal TTR mRNA levels in the choroid plexus differs between animals. Animals who are fairly independent soon after birth/hatching (e.g., chickens and sheep) are described as precocial and their brains are further developed at birth compared to altricial animals, whose brains are less developed at birth and are dependent on their mothers (e.g., rats, mice, marsupials). Precocial animals were found to have the peak of TTR mRNA in their choroid plexus before birth, whereas altricial animals had the peak of TTR mRNA in the choroid plexus after birth (58). For both groups of animals, the peak in TTR mRNA is just prior to the maximal growth rate in the brain. Given that the blood-brain barrier starts to develop when the first blood vessel grows into the brain (59) and that the choroid plexus develops faster than other parts of the brain, producing most of the CSF (and thus regulating its composition), and the peak of TTR mRNA just prior to the maximal growth rate of the brain, it follows that the choroid plexus-derived TTR could have a significant role in moving T4 from the blood into the CSF (57).

Whilst TTR is the only THDP known to be synthesized in the CNS (to the best of our knowledge, albumin and TBG are exclusively synthesized in the liver), this does not mean that albumin and TBG are absent from the CSF. In adult mammals, the protein concentration of the CSF is about 0.43 g/l compared to that in blood of about 70 g/l (60). Plasma proteins are present in the CSF at a concentration inversely proportional to their Stokes radius (61). Thus, whilst albumin is not synthesized in the CSF, it is present in the CSF; similarly for TBG in species where TBG is synthesized by the liver. It follows that, for example, although the choroid plexus of fish and amphibians do not synthesize TTR, their CSF would contain some albumin. Similarly, the CSF in reptiles and birds contains albumin and TTR; and the CSF of humans contains albumin, TTR, and TBG. However, the ratio of TTR to albumin in the CSF is very different to that in the blood: whereas in the blood albumin comprises ~50% total protein and TTR comprises ~0.4% total protein, in the CSF albumin comprises ~40% total protein and TTR comprises about 4% total protein (45). Thus, the TTR to albumin ratios and TH distribution kinetics would differ significantly. TTR is the main carrier of TH in the CSF (62, 63). Presumably, during the peak in TH concentration in blood during development, when the liver is synthesizing an “additional” THDP, some of that protein will enter the CSF. To date, it is unknown if TH levels in the CSF peak when (or soon after) the TH levels in the blood peak. Indeed, it might not be a reasonable question to consider, as the concentration of proteins and other molecules is not consistent throughout the CSF (46). In contrast to the blood, which mixes within minutes and is fairly homogeneous, the CSF flows in a directional “pipeline-like” manner and measurements of concentrations of its components differ depending on the sampling site [see (12)].

## Bioavailability of THs to Target Tissues

THs can exert both genomic and non-genomic actions. Regardless of which type of action a given TH will have, it is still required to get from its site of synthesis to its target tissue/cell and this is mediated via the THDPs: TTR, albumin, and TBG (depending on the species and stage of development).

Once THs have arrived at their target cell and have dissociated from the THDP, they are able to enter cells via TH transmembrane transporter proteins. These TH transmembrane transporters belong to the family of solute carriers and those known to move THs into and out of cells are the monocarboxylate transporters MCT8 and MCT10; L-amino acid transporters LAT1 and (depending on the species) LAT2; and organic anion transporter peptide OATP1C1 [for review see (64)]. Of these, only MCT8 and MCT10 are exclusive for the transmembrane transport of THs.

As mentioned above, the majority of TH secreted from the thyroid gland is in the form of T4 and around 80% of T3 is generated by local deiodination in target cells e.g., various regions of the brain produce differing proportions of T3 via local deiodination (65). Deiodination is carried out by a family of deiodinase enzymes, each of which can remove a specific iodine atom from a TH. Deiodinases can be classified by their broad reactions as either Outer Ring Deiodinases or Inner Ring Deiodinases, according to the position of the iodine atom being removed. Deiodinases can also be classified via their structures (amino acid sequences), locations and substrate preferences: Dio1, Dio2, and Dio3 [for review see (66)]. Deiodinases can either activate or inactivate THs within a cell. Whereas, genomic pathways are regulated mainly by T3, non-genomic pathways may be regulated by a greater number of TH derivatives (67).

## Impact of THDPs in Human Disease

Albumin, TTR, and TBG are negative acute phase plasma proteins (68) i.e., following stress, illness, surgery, or injury, their rates of synthesis in the liver decrease. This is thought to result in a transient increase in free TH in blood, which can then enter cells and direct anabolic reactions to restore health and homeostasis. Humans lacking either albumin (www.albumin.org) or TBG [see (69)] have been reported and were essentially without overt symptoms. Until now, no human lacking TTR has been reported. Could lack of TTR be incompatible with human life? Could this be due to TTR being the main protein synthesized and secreted by the choroid plexus or due to TTR being the main source of delivery of THs to tissues?

On the other hand, albumin and TBG have very few diseases associated with them: analbuminaemia [(70), www.albumin.org] and a variant of TBG in Australian Aborigines which has low affinity for THs (71), whereas TTR has major diseases associated with it: the various forms of TTR amyloidosis. Familial Amyloidotic Polyneuropathy (FAP) is a late onset autosomal dominant form of amyloidosis. More than 100 point mutations have been associated with causing TTR FAP (72). This is a significant number, as the polypeptide chain has only 127 amino acids in total (TTR is a homo-tetramer). In addition, wild type TTR can also form amyloid: Senile Systemic Amyloidosis. This occurs most frequently in the hearts of elderly men (73). The cause for wild type TTR to spontaneously form amyloid in the heart is currently unknown.

The first approved anti-TTR amyloid drug to come onto the market was Tafamidis (trade name Vyndaqel), a compound designed by Jeff Kelly and colleagues (74). Very recently, Patisiran (trade name ONPATTRO) has been approved by the European Commission and the United States Food and Drug Administration for the treatment of TTR amyloidosis. This is the first world-wide approved RNAi therapeutic! Apparently, patients receiving this RNAi therapy did not show deficiencies in thyroid or vitamin A metabolism. This could be due to only a small proportion of TTR circulating in blood having a TH or RBP-retinol bound.

An increasing body of knowledge is building around associations of altered TTR concentrations in the blood and/or CSF and a variety of diseases such as Alzheimer's Disease (75–77), rheumatoid arthritis (78, 79), schizophrenia (80, 81), preeclampsia (82–84), Guillain-Barre syndrome (85–87), and depression (88), and references in Alshehri et al. (41).

## Conclusion

Whether TH actions are via genomic or non-genomic pathways, THs need to get from their site of synthesis to their sites of action via THDPs in the blood and CSF. The network of THDPs is augmented during the developmental surge in THs in blood, providing increased distribution capacity. The THDPs have distinct profiles during both ontogeny and phylogeny, such that very fine regulation of free TH available to enter cells is highly controlled. In adult humans, lack of albumin or TBG are tolerated, yet a single point mutation in TTR can lead to disease. Humans lacking TTR have not yet been identified. The implication is that lack of TTR in humans is incompatible with life. This could be due to TTR being responsible for most delivery of TH to tissues or due to its role in moving TH from the blood into the CSF via the choroid plexus. Abnormal levels of TTR in humans are increasingly being associated with a variety of diseases. It is unknown if the altered TTR levels are a cause or a consequence of these diseases.

## Author Contributions

Overall structure was suggested by SJR. SAR and IG wrote most drafts with guidance from MP and NO. SJR edited the final version.

### Conflict of Interest Statement

The authors declare that the research was conducted in the absence of any commercial or financial relationships that could be construed as a potential conflict of interest.
